# Intraocular Neoplasm Suggested by Ocular POCUS Examination

**DOI:** 10.24908/pocusj.v11i01.19937

**Published:** 2026-04-22

**Authors:** Felipe Hernández-Restrepo, Santiago Hernández-Moreno, Alejandro Cardozo-Ocampo

**Affiliations:** Emergency Department, Fundación Instituto Neurológico de Colombia, Medellín, Colombia

**Keywords:** POCUS, Ocular POCUS, ER POCUS, Choroidal melanoma

## Abstract

We report the case of a 30-year-old woman with no relevant medical history who presented with a new-onset headache associated with nausea, vomiting, photophobia, phonophobia, and right nasal hemianopsia. Ocular point of care ultrasound (POCUS) examination revealed a hyperechoic lesion adhered to the vascular layer that did not move with eye movements. The patient was referred to ophthalmology, where choroidal melanoma was confirmed. Extension studies showed no metastases, and enucleation was performed. This case highlighted the importance of ocular POCUS in the diagnostic approach to this patient.

## Introduction

Intraocular neoplastic lesions are uncommonly diagnosed in emergency settings. However, neoplasms such as uveal melanoma can be catastrophic, with a 5-year survival rate of up to 30%, highlighting the importance of timely diagnosis [1,2]. While intraocular neoplastic lesions should be considered in patients with visual symptoms, other conditions such as stroke, acute glaucoma, or retinal detachment, are more common [3].

We report the case of a patient who presented with a headache and nonspecific ocular symptoms whose diagnosis was aided by ocular POCUS examination.

## Case Presentation

A 30-year-old woman with no significant medical history presented to the emergency department with a 2-day history of pulsatile occipital headache, associated with nausea, vomiting, photophobia, and phonophobia. She also reported right nasal hemianopsia but denied ocular pain. The symptoms did not improve with nonsteroidal anti-inflammatory drugs and acetaminophen. Of note, the patient did not typically suffer from headaches. On admission, her vital signs were normal and her neurological exam confirmed hemianopsia without focal deficits or other visual disturbances.

**Figure 1.**
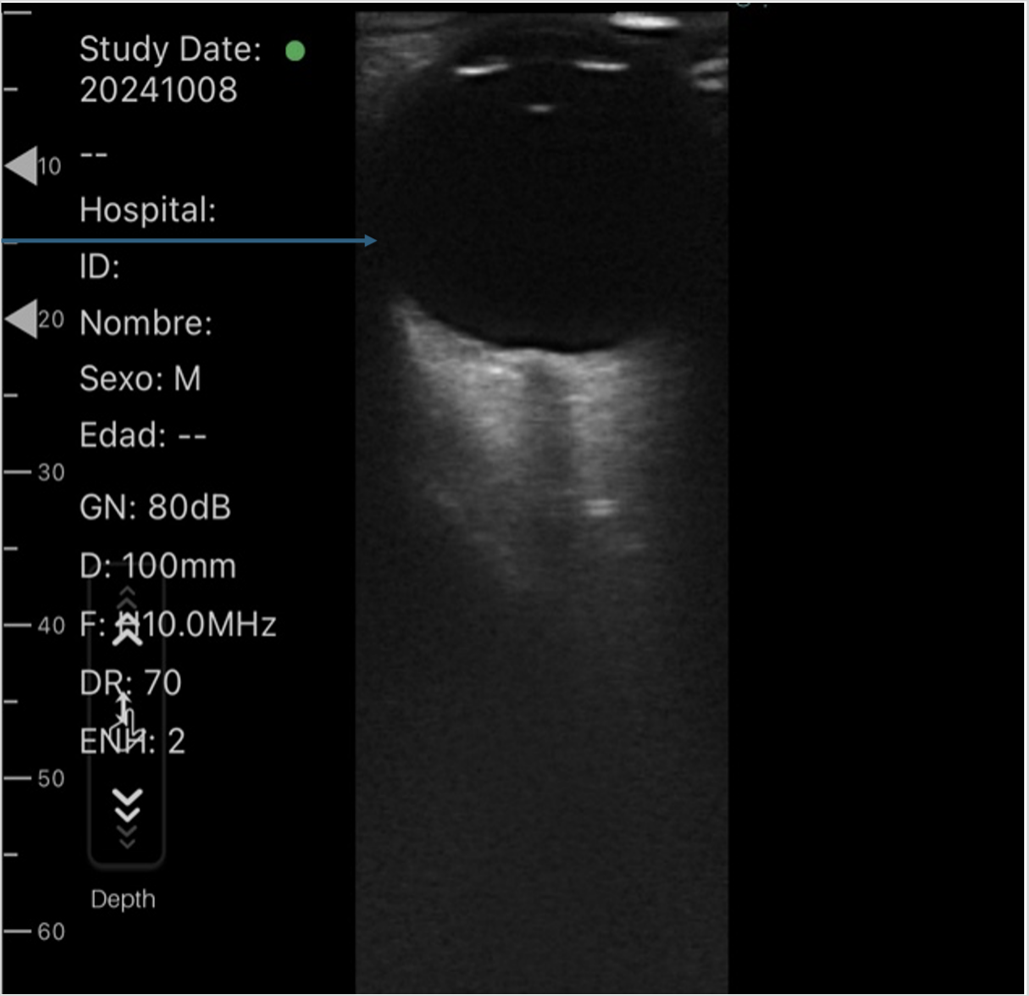
Ocular point of care ultrasound (POCUS) of the left eye. The blue arrow indicates a healthy left globe.

**Figure 2.**
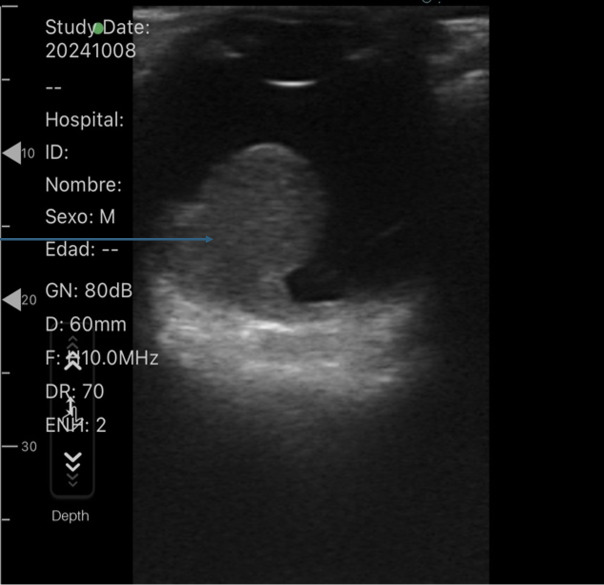
Ocular point of care ultrasound (POCUS) of the right eye The blue arrow points to a space-occupying lesion in the right globe.

**Figure 3.**
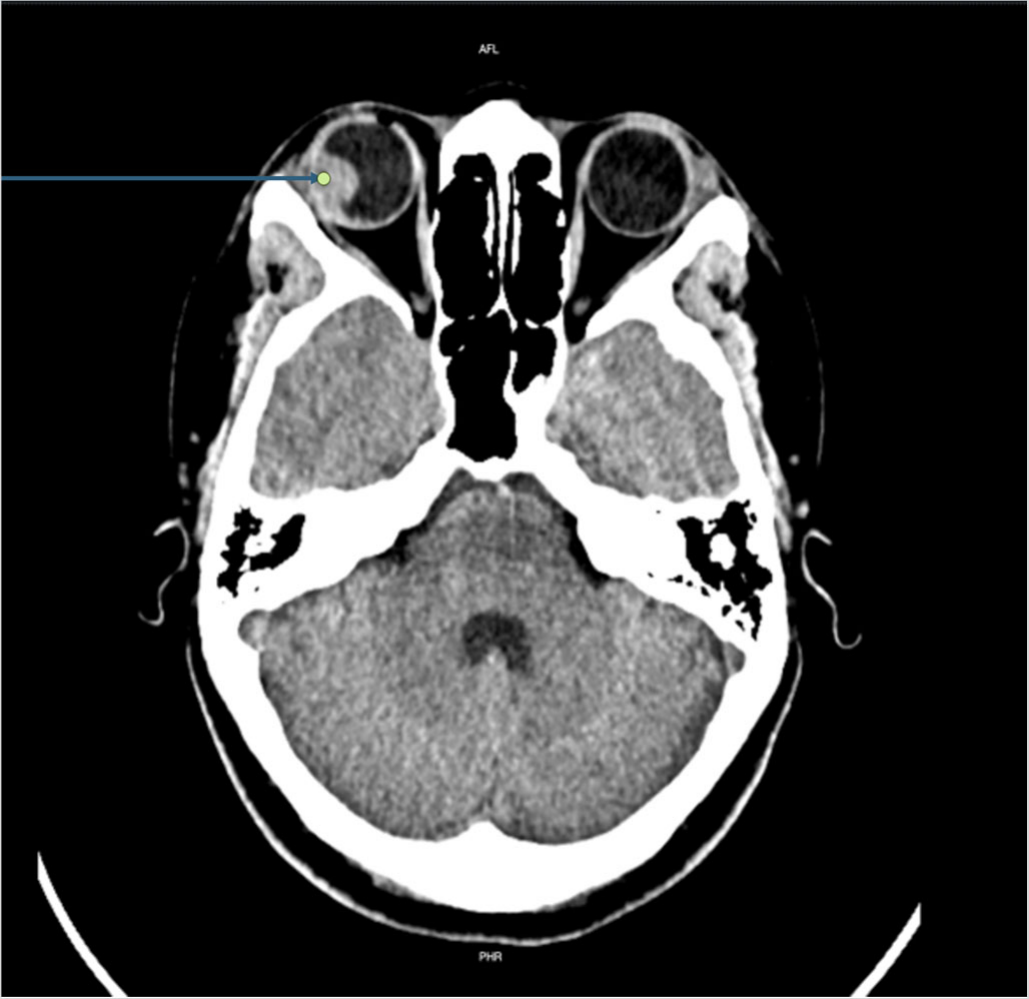
Non-contrast head computed tomography scan. The blue arrow indicates a space-occupying lesion in the right globe.

As our facility lacked an ophthalmology service, a comparative ocular POCUS was performed (Figures 1 and 2). This examination revealed a normal posterior chamber in the left eye but a hyperechoic lesion in the right eye, adhered to the vascular layer and non-mobile with eye movements. A non-contrast brain computed tomography (CT) scan was ordered, which showed no space-occupying lesions but confirmed the intraocular lesion (Figure 3).

Due to the lack of an ophthalmology service at our facility, the patient was referred to another institution. There, fundoscopic examination described a large, pigmented mass in the inferotemporal retina with orange pigment and surrounding subretinal fluid. Enucleation was proposed, and pathology confirmed a diagnosis of mixed choroidal melanoma.

## Discussion

Choroidal melanoma is a malignant lesion of the uveal tract. Despite its relative rarity, it is the most common ocular melanoma, occurring more frequently in younger women [1,4]. The clinical presentation for choroidal melanoma is usually nonspecific, which often leads to consideration of other differential diagnoses in the emergency department, such as acute glaucoma, stroke, or retinal detachment. A well-conducted physical exam and fundoscopic evaluation are critical for diagnosis. Ocular POCUS can complement the physical exam by evaluating the posterior chamber and ruling out common conditions such as retinal detachment, vitreous hemorrhage, lens dislocations, or, as in this case, ruling in a neoplastic lesion [4,5].

Ocular POCUS is relatively easy to perform because the eye's high liquid content provides an excellent medium for sound transmission. This allows detection of lesions that appear hyperechoic within the aqueous humor, as observed in this case. Some authors have suggested that the learning curve for emergency physicians is relatively short, as this examination is quick, easy to perform, and non-invasive [6].

Timely diagnosis is essential for intraocular neoplastic lesions as they are highly invasive, with a predilection for the liver and skin. Enucleation and chemotherapy are the main treatments to prevent progression and/or metastatic dissemination [7].

## Conclusion

Intraocular neoplastic lesions are rare in emergency settings. For patients with nonspecific symptoms, physical examination can be supplemented by ocular POCUS, which can suggest neoplasia when non-mobile solid lesions are detected. In this case, ocular POCUS contributed to the appropriate management of the patient.
